# Immobilized Hydroxymethylfurfural Oxidase Enables Robust Biocatalytic Production of 2,5-Furandicarboxylic Acid from Crude 5‑Hydroxymethylfurfural

**DOI:** 10.1021/acssuschemeng.5c13549

**Published:** 2026-04-04

**Authors:** Darly Concha, Garazi Ortiz-Orruño, Marina Guillén, Oscar Romero, Kírian Bonet-Ragel

**Affiliations:** Bioprocess Engineering and Applied Biocatalysis Group, Department of Chemical, Biological and Environmental Engineering, Universitat Autònoma de Barcelona, 08193 Bellaterra, Spain

**Keywords:** biocatalysis, 2,5-furandicarboxylic acid (FDCA), 5-hydroxymethylfurfural (HMF), enzyme immobilization, hydroxymethylfurfural oxidase (HMFO), cellulose-based supports, sustainable plastics

## Abstract

Bioplastics such as poly­(ethylene 2,5-furandicarboxylate) (PEF), synthesized from biobased 2,5-furandicarboxylic acid (FDCA), offer a promising alternative to petrochemical-derived plastics. Enzymatic conversion of 5-hydroxymethylfurfural (HMF) to FDCA via hydroxymethylfurfural oxidase (HMFO) is environmentally benign but is limited by catalyst stability. This study addresses these challenges through a high-performance biocatalytic platform based on the one-step purification and immobilization of the engineered enzyme 8BxHMFO fused with a carbohydrate-binding module (CBM3). Utilizing the high affinity of CBM3 for microcrystalline cellulose (Perloza MT100), the biocatalyst achieved a significant increase in thermal stability (*T*_m_ = 53.2 °C) and enhanced resistance to oxygen interfacial inactivation. The system’s efficacy was demonstrated with pure HMF (>95% conversion; 7.2 g·L^–1^ FDCA), and its robustness was validated using a 15% (w/w) crude extract. The biocatalyst maintained its catalytic performance in the presence of minor matrix components, exhibiting a highly competitive FDCA yield (70.6%) and titer (5.7 g·L^–1^). FDCA recovery was achieved with 72% efficiency using a mild ethanol-based extraction process. These findings and sustainable indicators validate the robustness of the system under realistic conditions, demonstrating its potential as a sustainable platform for FDCA production and contributing to broader PEF bioplastic adoption.

## Graphical abstract

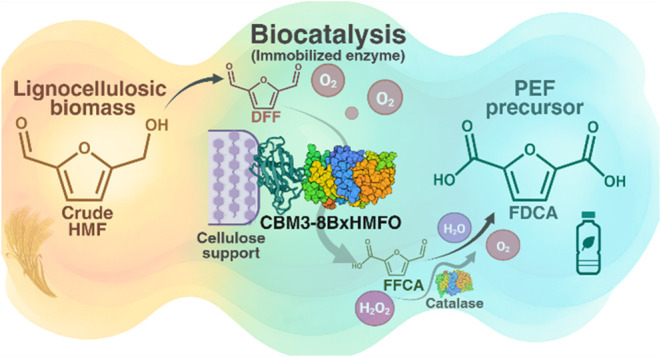

## Introduction

The widespread use of plastic materials has generated severe environmental consequences. Among them, polyethylene terephthalate (PET) contributes substantially to plastic pollution in terrestrial and marine ecosystems.^1–3^ The current recycling technologies, which remain environmentally detrimental, underscore the urgency of developing sustainable alternatives.^4,5^ In response to these sustainability challenges, poly­(ethylene 2,5-furandicarboxylate) (PEF) has emerged as a promising biobased alternative to PET. PEF is synthesized from renewable resources and utilizes 2,5-furandicarboxylic acid (FDCA) as a monomer, replacing terephthalic acid in conventional polyesters.^6–8^

FDCA can be produced via the selective oxidation of 5-hydroxymethylfurfural (HMF), a platform chemical obtained from lignocellulosic biomass. Traditional chemical oxidation routes, such as noble metal or transition metal oxide catalysis, require harsh reaction conditions, elevated temperatures, and toxic solvents, leading to undesirable byproducts and high processing costs.^9–11^ Recent advances using heterogeneous catalysts, including Co-based oxides or microwave-assisted noble metal systems, have improved activity and energy efficiency but still rely on chemical oxidants and pressurized conditions.^12–14^ In contrast, enzymatic oxidation offers an environmentally benign alternative, operating under mild conditions, employing molecular oxygen as a clean oxidant, and delivering high selectivity toward FDCA.^15,16^

Enzymatic oxidation of HMF frequently relies on multienzymatic cascades, which complicates process optimization. Nevertheless, hydroxymethylfurfural oxidase (HMFO), a flavoprotein oxidoreductase belonging to the glucose-methanol-choline (GMC) enzyme superfamily, exhibits notable substrate promiscuity, catalyzing the oxidation of a broad range of furanic compounds, including the primary alcohol oxidation of HMF.^17,18^ These enzymes contain flavin adenine dinucleotide (FAD) as a tightly bound prosthetic group, which is essential for their redox activity.^18–20^

Native HMFOs, such as those from *Methylovorus* sp. MP688, demonstrate high catalytic efficiency in oxidizing both alcohol and aldehyde functional groups.^18,19^ The enzyme catalyzes the full oxidation of HMF to FDCA through a sequential three-step process that includes the formation of intermediate compounds: 2,5-diformylfuran (DFF) and 5-formylfuran-2-carboxylic acid (FFCA). Advances in protein engineering have enabled the development of improved HMFO variants, such as 8BxHMFO, capable of catalyzing complete oxidation of HMF into FDCA, exhibiting enhanced thermal stability and reduced susceptibility to oxidative deactivation position them as promising candidates for industrial-scale biocatalytic applications.^21,22^ Nevertheless, their practical implementation is still hampered by challenges such as poor stability at gas–liquid interfaces, product inhibition, particularly associated with hydrogen peroxide accumulation, and low expression yields in recombinant hosts.^18,22,23^ To overcome these limitations, recent efforts have focused on rational enzyme design and fine-tuning of reaction parameters to enhance overall catalytic efficiency and robustness.^20^

However, HMFOs still face challenges in operational stability due to their oxidative mechanism, which necessitates the utilization of molecular oxygen as the terminal electron acceptor. The use of molecular oxygen as an electron acceptor leads to local oxygen depletion and the formation of reactive oxygen species (ROS), which may induce enzyme deactivation. Furthermore, the gas–liquid interface in aerated bioreactors promotes protein denaturation via interfacial adsorption and aggregation.^23,24^ The high surface tension at these boundaries exerts mechanical stress on the protein’s tertiary structure, triggering the exposure of hydrophobic cores and subsequent irreversible aggregation.^24^ To overcome these limitations, enzyme immobilization has been widely adopted as a strategy to improve catalytic stability, enable enzyme reuse, and facilitate integration into continuous processes.^25,26^ Enzyme immobilization is a well-established technique that improves catalytic stability, enhances resistance to denaturation, and enables application in multienzyme cascade systems.^27^ Additionally, immobilization facilitates enzyme recovery and reuse, offering a cost-effective and sustainable solution.^25^

In particular, immobilization via carbohydrate-binding modules (CBMs) offers a sustainable and efficient platform. This approach aligns with the principles of green chemistry by enabling enzyme purification and immobilization in a single step using renewable supports, such as microcrystalline cellulose. These matrices include Avicel PH-200, characterized by its irregular particle morphology and high crystallinity, as reported in previous studies,^28^ and Perloza MT100, which exhibits a uniform spherical architecture of porous regenerated cellulose. This support belongs to a family of matrices utilized in high-performance biotechnological applications, including the development of immunoaffinity adsorbents for the isolation of specific autoantibodies.^29^

CBM3 from *Clostridium thermocellum* binds specifically to crystalline cellulose and allows site-directed immobilization.^28,30^ This specific interaction, provides a superior alternative to traditional affinity systems such as Ni-NTA resins, which are often cost-prohibitive for large-scale applications due to expensive ligands and metal leaching risks.^28^ Furthermore, unlike nonspecific covalent grafting, which often results in random protein orientation and active site blockage,^31^ the CBM-mediated approach ensures that the enzyme remains correctly oriented and fully accessible, maximizing the biocatalytic performance.^32^ It also allows one-step purification and immobilization through specific carbohydrate interactions, providing a sustainable and cost-efficient alternative to traditional immobilization based on chemical functionalization.^28,33^ These developments underscore the increasing relevance of HMFOs in industrial biotechnology, playing a central role in advancing sustainable polymer production.^19,22,34,35^

Most biocatalytic studies for FDCA production have employed pure HMF as the substrate, which does not reflect the complexity of real biomass-derived hydrolysates. Crude HMF streams often contain impurities such as humins, organic acids, and residual sugars that may inhibit enzyme activity or interfere with downstream processing.^36,37^ Therefore, it is essential to validate the performance of biocatalysts under these more industrial/biorefinery relevant conditions to ensure their feasibility for industrial implementation.

In this study, we report the expression of the engineered 8BxHMFO enzyme fused to CBM3, its immobilization on a microcrystalline cellulose supports, and its application in the biocatalytic production of FDCA. To enhance enzyme stability against thermal and chemical stress, the immobilized biocatalyst was further cross-linked with glutaraldehyde, creating a robust heterogeneous system with increased structural integrity. The catalytic performance was evaluated using both pure HMF and crude HMF. This work applied an intensification strategy integrating one-step purification and immobilization, an optimized oxygen supply, and the evaluation of multiple HMF concentrations under previously established pH and temperature conditions. The results contribute to the integration of biocatalytic systems into circular biorefinery schemes for biobased polymer manufacturing, highlighting the system’s adaptability to realistic feedstocks and alignment with sustainability goals.

## Methods

### Materials

5-Hydroxymethylfurfural (HMF), 2,5-diformylfuran (DFF), 5-formyl-2-furancarboxylic acid (FFCA), and 2,5-furandicarboxylic acid (FDCA) were purchased from TCI Chemicals (Japan). 5-Hydroxymethylfurfural (Crude HMF) was obtained from AVA Biochem (Switzerland; Item Code A888.0883, Lot-No. 1812A-181213-RO1B-C-12). Flavin adenine dinucleotide (FAD) was obtained from Sigma-Aldrich (St. Louis, MO, USA). Microcrystalline cellulose Perloza MT100 was provided by Perloza s.r.o. (Czech Republic). Avicel PH-200 microcrystalline cellulose sample was kindly donated by DuPont N&B (New York, NY, USA).

### Enzymes

An octuple mutant variant of HMF oxidase (8BxHMFO; I73 V, H74Y, G356 K, V367R, T414 K, A419Y, A435E, and W466F) from *Methylovorus* sp. was genetically fused to a type 3 cellulose-binding module (CBM3) from *C. thermocellum*. The fusion was mediated by a flexible 12-amino acid linker to ensure the structural and functional autonomy of both protein domains.^28^ The synthetic gene was obtained from GenScript (USA) and cloned into the pVEF expression vector. Complete amino acid sequences for 8BxHMFO, CBM3, and the linker peptide are provided in the Supporting Information. The fusion enzyme was expressed in an auxotrophic M15-derived *Escherichia coli* strain, cultivated in high cell density cultures in a 5 L fed-batch reactor using an antibiotic-free defined medium.^38^ Cell disruption was performed at 1.6 kbar using a high-pressure disruptor (Constant Systems cell disruptor), and the enzyme was recovered in the soluble fraction of the lysate, yielding a specific activity of 14.8 ± 0.7 U·gDCW^–1^, a volumetric activity of 1209.5 ± 59.3 U·L^–1^, and an enzyme concentration of 2762.3 ± 126.5 mg·L^–1^. This corresponded to a specific activity of 0.44 ± 0.03 U·mg^–1^. Catalase was purchased from Sigma-Aldrich (St. Louis, MO, USA) and used as auxiliary enzyme.

### HMFO Activity Assay

The oxidase activity of CBM3–8BxHMFO was measured by monitoring oxygen consumption using a Pyro Science robust oxygen probe coupled to a FireSting-PRO oxygen meter. Reactions were performed in 2.5 mL of 50 mM Tris-HCl buffer (pH 8.0) at 30 °C with 300 rpm agitation. Enzymatic reactions were initiated by adding the enzyme (soluble and immobilized) and 60 mM HMF. One unit of enzymatic activity was defined as the amount of enzyme required to consume 1 μmol of molecular oxygen per minute under these conditions.

### One-Step Purification and Immobilization of CBM3–8BxHMFO

The one-step purification and immobilization procedure was carried out by incubating 9 mL of lysate containing CBM3–8BxHMFO with 1 g of Perloza MT100 or Avicel PH-200 at room temperature under gentle rotation using a roller mixer for 30 min. Enzyme loading varied between 0.65 and 4 U per gram of support. The biocatalyst was washed with a 1:20 solid-to-liquid ratio of 50 mM Tris-HCl buffer pH 8 and recovered by vacuum filtration.

A postimmobilization treatment was applied to the biocatalyst using different concentrations of glutaraldehyde solutions ranging from 0.1% to 1% (v/v) in 50 mM Tris-HCl buffer (pH 8.0), using a 1:10 solid-to-liquid ratio. The suspension was incubated for 30 min under gentle agitation, filtration, and extensive washing with the same buffer. The resulting cross-linked biocatalyst was stored at 4 °C until further use.

Immobilization was evaluated by determining the immobilization yield (IY%), recovery of catalytic activity (RCA%), retained activity (RA%), protein immobilization yield (protein %), and protein loading (mg·g_support_^–1^).^28^

1 $$i\mathrm{mmobilization}\;\mathrm{yield}\;\left(\mathrm{IY}\%\right):\;\left(\frac{A_{i}-A_{sn}}{A_{i}}\right)\times 100$$

2 $$r\mathrm{ecovery}\;\mathrm{activity}\;\left(\mathrm{RCA}\%\right):\left(\frac{A_{d}\cdot M_{\mathrm{support}}}{A_{i}\cdot V}\right)\times 100$$

3 $$r\mathrm{etained}\;\mathrm{activity}\;\left(\mathrm{RA}\%\right):\left(\frac{A_{\mathrm{sus}}-A_{\mathrm{sn}}}{A_{i}-A_{\mathrm{sn}})}\right)\times 100$$

4 $$i\mathrm{mmobilization}\;\mathrm{yield}\;\left(\mathrm{protein}\;\%\right):\left(\frac{P_{i}-P_{sn}}{P_{i}}\right)\times 100$$

5 $$i\mathrm{mmobilized}\;\mathrm{protein}\;\mathrm{per}\;g\;\mathrm{support}:\frac{\left(p_{i}-p_{\mathrm{sn}}\right)\cdot V}{M_{\mathrm{support}}}$$

6 $$p\mathrm{urification}\;\mathrm{factor}:\left(\frac{E_{\mathrm{support}}}{E_{\mathrm{offered}}}\right)$$

where *A*_i_: initial enzyme activity offered (U·mL^–1^); *A*_sn_: remaining enzyme activity measured in the supernatant (U·mL^–1^); *A*_sus_: enzyme activity of the biocatalyst suspension (U·mL^–1^); *A*_d_: enzyme activity of the biocatalyst obtained (U·g^–1^); *M*_support_: mass of the support used in the process (g); *V*: total volume of the immobilization solution (mL); *P*_i_: initial protein concentration (mg·mL^–1^); *P*_sn_: supernatant protein concentration (unbound proteins) (mg·mL^–1^); *E*_support_: concentration of the target enzyme immobilized on the support (mg·mL^–1^); and *E*_offered_: concentration of the target enzyme in the initial lysate (mg·mL^–1^).

### Protein Concentration

Protein concentration in soluble and immobilized fractions was quantified using the Bradford assay (Coomassie Protein Assay Reagent Kit). Briefly, 7 μL of sample was mixed with 200 μL of the reagent and incubated at room temperature for 10 min. Absorbance was recorded at 595 nm using a SPECTROstar Nano plate reader (BMG LABTECH GmbH, Germany). A bovine serum albumin (BSA) calibration curve ranging from 0 to 2 mg·mL^–1^ was used as a standard.

### Confocal Laser Scanning Microscopy

The immobilized CBM3–8BxHMFO enzyme on microcrystalline cellulose (Perloza MT100) was analyzed by fluorescence confocal microscopy. No external fluorescent labeling was required since the FAD cofactor of HMFO exhibits intrinsic autofluorescence. A suspension of the immobilized biocatalyst was prepared in 50 mM Tris-HCl buffer (pH 8.0) and diluted (1:100). Samples were examined using a Zeiss LSM confocal microscope with excitation at 405 nm and emission collection between 408 and 551 nm, corresponding to the autofluorescence spectrum of FAD. Confocal images were initially processed using ImageJ (FIJI), and further visualization and analysis were carried out with Imaris Viewer (version 10.2.0).

### Thermostability Analysis

Thermal stability of the soluble and biocatalyst was evaluated by determining the melting temperature (*T*_m_) using SYPRO Orange dye. Samples (22.5 μL) were mixed with 2.5 μL of SYPRO dye in a 96-well plate. The melt curve was recorded from 10 to 95 °C in 0.5 °C increments using the CFX96 Touch Real-Time PCR Detection System (Bio-Rad Laboratories), with fluorescence measurements acquired at each temperature interval.

### FDCA Production Using Immobilized CBM3–8BxHMFO

The oxidation of HMF was conducted in a basket-type bioreactor using immobilized CBM3–8BxHMFO. This configuration was specifically selected to prevent the flotation and adhesion of the biocatalyst (immobilized enzyme on a microcrystalline cellulose support) to the reactor walls caused by continuous aeration, ensuring that the entire biocatalyst load remained submerged and catalytically active throughout the process. The reactions were performed by using the biocatalyst that had been previously immobilized on Perloza MT100 and cross-linked with 0.25% glutaraldehyde, resulting in a protein loading of approximately 50 mg per gram of support. Reactions were carried out in 50 mM Tris-HCl buffer (pH 8.0) containing various concentrations of HMF (6–125 mM), catalase was supplemented every 24 h (97.7 U·mL^–1^) following the stability profile observed in Figure S2, and continuous air bubbling at a flow rate of 0.6 vvm. Temperature and agitation were maintained at 30 °C and 300 rpm, respectively. pH was monitored and controlled using 1.5 M NaOH and a Metrohm 916 Ti-Touch titrator. Samples were withdrawn at defined time intervals and analyzed via HPLC.

### HPLC Analysis

Quantification of HMF and its oxidation products (FDCA, DFF, and FFCA) was performed using a 1220 Infinity Agilent HPLC system equipped with a 1260 Infinity II Agilent UV detector. Prior to analysis, samples were diluted to a final concentration of 1 mM by using the mobile phase. Separation was achieved with a SUPELCOGEL C-610H column using 5 mM H_2_SO_4_ as the mobile phase at a flow rate of 0.5 mL·min^–1^ and 30 °C column temperature. Detection was carried out at 264 nm.^22^ The retention times for each compound were 51.3 min for HMF, 64.6 min for DFF, 37.4 min for FFCA, and 26.2 min for FDCA.

For the comprehensive chemical characterization of technical-grade crude HMF (including the quantification of organic acids and minor furanic impurities), specific chromatographic methods were used. Full methodological details and equipment specifications for these analyses are described in the Supporting Information.

### FDCA Purification

FDCA purification was performed by acid-induced precipitation, followed by sequential washing and solvent extraction. Following the biocatalytic reactions, hydrochloric acid was added dropwise to the reaction mixture until the pH reached 0, promoting complete precipitation of FDCA. The resulting precipitate was collected by vacuum filtration and initially washed with 100 mM HCl to remove soluble impurities. The solid was subsequently subjected to three additional washes with acidified water to ensure the removal of residual salts and unreacted substrates.

To enhance purity, the FDCA precipitate was dissolved into three volumes of ethanol (3 × 17.8 mL) under agitation, and the resulting suspension was filtered to eliminate ethanol-insoluble impurities. The ethanol phase containing FDCA was then concentrated by rotary evaporation at 40 °C until it reached dryness. The final solid product was stored at room temperature until further analysis. The purity of the recovered FDCA was determined by comparing the concentration measured via HPLC to the total mass of the powder dissolved (gravimetric basis). The purification workflow is summarized in Figure S10.

### Sustainable Indicators

To provide a robust quantitative assessment of the environmental performance, the *E*-factor and Global Warming Potential (GWP) were utilized as standard indicators of waste minimization and carbon footprint, respectively. Briefly, the *E*-factor is a direct indication of the amount of waste generated by the process per unit of the product of interest. GWP is a metric that quantifies the total amount of CO_2_ emitted by a process per kilogram of the product. This metric may be calculated in two ways. First, it may be calculated as GWP_energy_, which is the GWP that results from the energy used in the reaction. Second, it may be calculated as GWP_mass_, which is the GWP that results from the resources consumed and disposed of during the process.^39^ Equations used for GWP calculations and *E*-factor:

GWP_mass

7 $$\mathrm{GWP}\left(\mathrm{water}\left(\mathrm{wwtp}\right)\right)=\frac{0.073\cdot \%\mathrm{watertreated}}{yield\cdot \left[SL\right]}$$

where %watertreated is the % of water that will be treated in wwtp (i.e., not reused), yield is the yield of the reaction (%), and [SL] is the substrate loading in kg·L^–1^.

GWP_energy

8 $$\mathrm{GWP}\left(\mathrm{water}\left(\mathrm{energy}\right)\right)=\left(\frac{0.037\cdot \Delta T}{\mathrm{yield}\cdot \left[\mathrm{SL}\right]}\right)+t\cdot \left(\frac{0.0056\cdot \Delta T}{\mathrm{yield}\cdot \left[\mathrm{SL}\right]}\right)$$

where yield is the yield of the reaction (%), [SL] is the substrate loading in kg·L^–1^, and Δ*T* is the temperature difference from 20 °C and the temperature of the system.

*E*-factor

9 $$E‐\mathrm{factor}=\frac{\mathrm{mass}\;\mathrm{of}\;\mathrm{total}\;\mathrm{waste}\;\left(\mathrm{kg}\right)}{\mathrm{mass}\;\mathrm{of}\;\mathrm{total}\;\mathrm{product}\;\left(\mathrm{kg}\right)}$$

## Results and Discussion

### Enzyme Immobilization on Microcrystalline Cellulose

FDCA synthesis using soluble 8BxHMFO, with air bubbling to supply molecular oxygen, led to rapid enzyme inactivation with the enzyme retaining 20% of its initial activity after 52 h (Figure S1). While bubbling air into the reaction medium is a common strategy for oxygen supply, it is well documented that interfacial stress at the gas–liquid interface can lead to enzyme denaturation,^40^ a phenomenon also observed for HMFO.^23^ This phenomenon, attributed to unfolding, aggregation, and loss of enzymatic activity, is primarily caused by dynamic gas–liquid interfaces and mechanical agitation.^24^ These findings underscore the importance of enzyme stabilization strategies, such as immobilization, especially for applications involving gaseous substrates. Immobilization can provide a protective microenvironment that shields the enzyme from interfacial stresses at gas–liquid boundaries, thereby reducing the risk of denaturation and maintaining catalytic activity over prolonged reaction times.

To enable an efficient, cost-effective, and sustainable immobilization strategy, we genetically fused the 8BxHMFO enzyme with CBM3, a carbohydrate-binding module known for its strong affinity toward cellulose, via a flexible peptide linker. This fusion facilitated a one-step purification and immobilization process onto microcrystalline cellulose, an attractive support material due to its cost-effectiveness, renewability, and sustainability. As outlined in the previous section, two different types of microcrystalline cellulose supports were evaluated: Avicel PH-200, characterized by its irregular particle morphology, porosity, and high crystallinity, and Perloza MT100, which presents a uniform spherical structure of porous regenerated cellulose matrix.

The immobilization process was completed within 30 min in both cases, as confirmed by the total depletion of the enzymatic activity in the supernatant (Figure 1A). This indicates the quantitative partitioning of functional CBM3–8BxHMFO onto the support, while the residual bands of similar molecular weight observed in the SDS-PAGE (Figure S4A) are attributed to endogenous *E. coli* host proteins. SDS-PAGE analysis (Figure 1B) corroborated the efficient enrichment of the target protein. Lane 2 corresponds to the total cell lysate, while lane 3 represents the biocatalyst on Avicel PH-200, showing a prominent band consistent with the expected molecular weight of CBM3–8BxHMFO. Similarly, lanes 4 and 5 represent the lysate and biocatalyst on Perloza MT100, respectively, both confirming successful immobilization through the appearance of the target protein band. The strategy achieved a purification factor of 22.5 for Avicel PH-200 and 19.8 for Perloza MT100, underscoring the efficiency of CBM3-mediated immobilization as a one-step strategy for purification and immobilization. These values reflect a highly efficient capture of the target enzyme from crude lysates and validate the use of CBMs as functional affinity tags for biocatalyst preparation. Comparable findings have been reported in previous studies employing CBM fusions. Full recovery and immobilization of GroEL-CBD onto microcrystalline cellulose directly from cell lysate has been demonstrated, emphasizing the cost-efficiency of this method.^41^ The capacity of CBM-tagged enzymes to bind selectively to cellulosic matrices, enabling enzyme recovery without prior purification, has also been highlighted.^32,42^ Furthermore, purification factors between 3 and 10.1 were reported for CBM-tagged alcohol dehydrogenases.^28^ Altogether, these results confirm that CBM-based immobilization is not only technically robust but also strategically advantageous, as it integrates purification and immobilization, thereby significantly reducing downstream processing costs, which can constitute 50–80% of total manufacturing expenses.^43–45^

**Figure 1:**
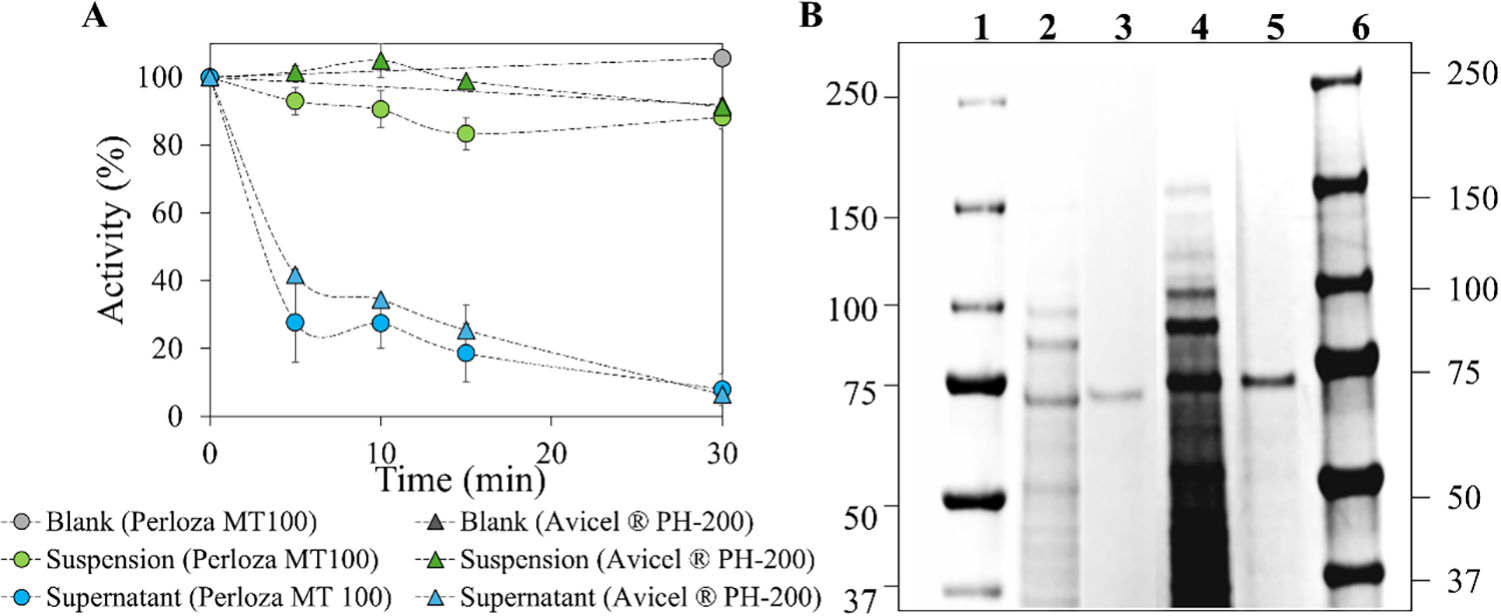
(A) Immobilization kinetics of CBM3–8BxHMFO in Perloza MT100 (dots) and Avicel PH-200 (triangles) Supports. (B) SDS-PAGE: lanes 1 and 6 MWM (kDa); (CBM3–8BxHMFO, MW: 75.1 kDa) lanes 2 and 3: blank (Total lysate) and biocatalyst onto Avicel PH-200; lanes 4 and 5: blank (Total lysate) and biocatalyst onto Perloza MT100.

Table S1 summarizes the key performance indicators of the one-step purification immobilization process. A high immobilization yield and recovery activity of 93 and 27.2% was obtained for Avicel PH-20, and 92 and 28.8% for Perloza MT100, respectively, at a protein loading of 50 mg g^–1^. At such high surface densities, the expressed activity is limited by the high amount of biocatalyst and steric hindrance, which reduce the conformational flexibility of the enzyme,^46^ as well as by pronounced mass transfer and internal diffusion limitations.^46,47^ Under these conditions, the dense packing of the enzyme on the cellulose surface hinders the diffusion of HMF to the active sites, as well as the release of reaction products, making diffusion the rate-limiting step and resulting in low recovered activities.^46,47^ Both supports exhibited a high protein loading capacity of 50 mg/gram of support. These outcomes are consistent with previous reports employing various microcrystalline cellulose matrices such as Avicel PH-101, regenerated amorphous cellulose (RAC), and bacterial nanocellulose which have demonstrated similar or slightly lower adsorption capacities.^28,41,42,48^ The high immobilization efficiency and rapid binding kinetics observed in this study support the effectiveness of CBM3-based affinity immobilization as a robust one-step purification strategy. The low-cost process and renewable nature of the support highlight its industrial relevance, particularly in the context of sustainable biocatalyst design. Such an approach directly aligns with the principles of circular economy, leveraging low-cost agro-industrial residues and enhancing the environmental sustainability of bioprocesses.^28,30^ Furthermore, unlike microcrystalline cellulose, Perloza MT100 consists of spherical beads of porous regenerated cellulose. Parallel experiments within our group confirmed its superior mechanical stability and pressure resistance, identifying it as the optimal support for FPLC column applications. Based on these results, Perloza MT100 was selected as the primary support for further biocatalytic evaluation.

The first attempt at FDCA production using CBM3–8BxHMFO immobilized on a Perloza MT100 showed a progressive loss of enzyme activity (Figure S3). This phenomenon was attributed to the desorption of the enzyme from the cellulose matrix, which released the enzyme into the solution, where it was likely denatured (Figure S4 and Table S2). This outcome is consistent with recent studies demonstrating that CBM–cellulose interactions, particularly those involving CBM3, are dynamic and reversible, with possible elution in the presence of glucose or low-polarity solvents.^30,49^

To improve retention and enhance operational stability, glutaraldehyde was employed as a cross-linking agent following the initial immobilization stage. This bifunctional reagent can form covalent bonds between enzyme molecules. Its effectiveness in stabilizing biocatalyst by reinforcing their structural conformation is well supported in the literature.^31,50^

Figure 2A shows that cross-linking with glutaraldehyde 0.25% resulted in a moderate reduction in recovered enzymatic activity to 20%. This decrease suggests a partial restriction of enzyme flexibility, possibly impairing substrate access to the active site. Notably, in the absence of glutaraldehyde, only 30% of the initial activity was recovered, which can be attributed to the high protein loading on the support estimated at nearly 50 mg of protein per gram of support. Such high surface occupancy may promote a hinder substrate diffusion, leading to limited catalytic performance.^51^ At higher concentrations (1%), catalytic activity was almost completely reduced, indicating that excessive cross-linking severely hinders enzymatic activity.

**Figure 2:**
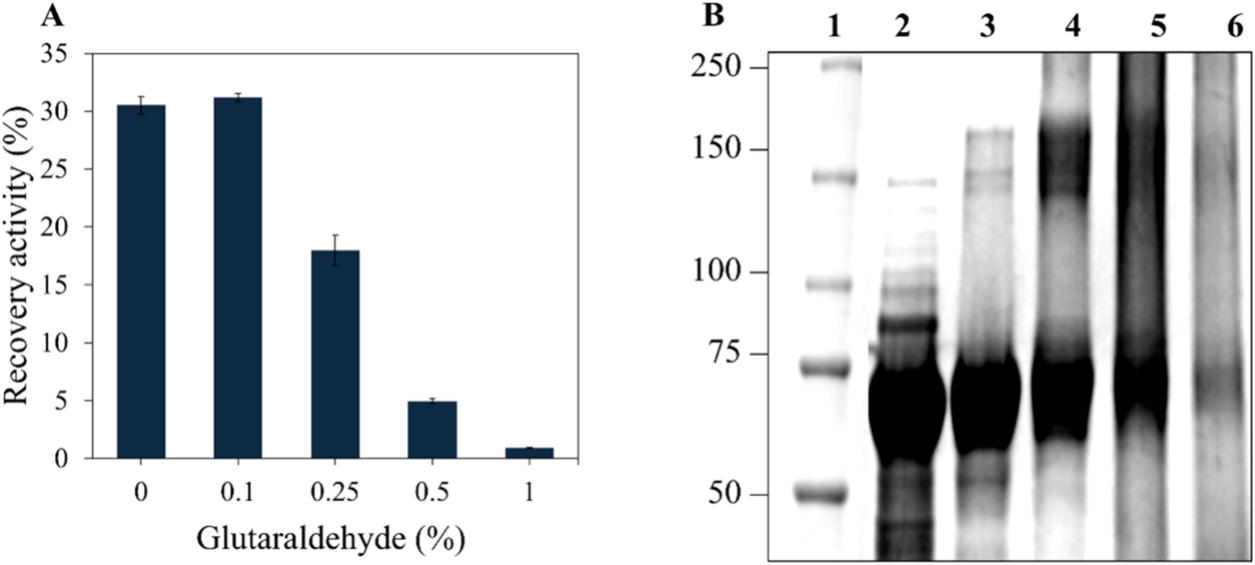
(A) Recovery activity of the different concentrations of glutaraldehyde used as cross-linker. (B) SDS-PAGE analysis of the supernatants collected from the cross-linked biocatalyst to evaluate enzyme leaching, where lane 1 is the molecular weight marker in kDa; (CBM3–8BxHMFO, MW = 75.1 kDa). Lanes 2 to 6 show the supernatant fractions obtained after cross-linking the biocatalyst with 0, 0.1, 0.25, 0.5, 1% glutaraldehyde, respectively.

SDS-PAGE analysis of the supernatants collected from the cross-linked biocatalyst was performed to evaluate enzyme leaching (Figure 2B) and confirmed enhanced enzyme retention on the support, as demonstrated by the progressive disappearance of the soluble enzyme band with increasing glutaraldehyde concentration. This pattern reflects stronger covalent bonding between the enzyme and among enzyme molecules, improving immobilization stability. However, this enhanced retention came at the cost of catalytic performance. At 0.25% glutaraldehyde, recovered enzymatic activity dropped to 20%, suggesting a trade-off between retention and catalytic functionality. Notably, high concentrations of glutaraldehyde (e.g., 1%) resulted in near-complete inactivation, consistent with previous reports describing enzyme deactivation due to excessive cross-linking.^52–55^ While low glutaraldehyde concentrations have been shown to preserve catalytic function and reduce leaching,^52,55^ the 0.25% cross-linked biocatalyst was selected for subsequent experiments as it offered the most balanced condition between operational stability and catalytic activity, effectively minimizing enzyme leaching while retaining significant functionality compared to higher cross-linker concentrations.

### Characterization of Immobilized Biocatalyst: Thermal Stability and Microscopic Distribution

To evaluate the impact of immobilization and cross-linking on the structural stability of CBM3–8BxHMFO, the melting temperature (*T*_m_) was measured for the soluble enzyme, its immobilized form, and the glutaraldehyde cross-linked biocatalyst (Table S3). The biocatalyst exhibited a *T*_m_ of 53.2 °C, which represents a significant increase from the *T*_m_ of the soluble counterpart (49.7 °C). This enhancement in thermal stability is likely attributable to the conformational restrictions imposed by the covalent and noncovalent interactions with the cellulose matrix, which can limit the flexibility of the polypeptide chain and reduce the likelihood of thermal unfolding.^56–58^ Such stabilization has been widely observed for biocatalysts and is particularly relevant for biocatalytic applications requiring elevated operational temperatures. The observed increase in *T*_m_ further supports the role of immobilization not only in facilitating enzyme reuse but also in enhancing resistance to thermal denaturation, a desirable trait for long-term processing under industrial conditions.

Conversely, increasing the concentration of glutaraldehyde beyond the optimal range led to a slight but consistent decrease in the *T*_m_ values. This observation, consistent with previous reports, suggests that excessive cross-linking can introduce localized structural stresses or distortions, disrupting the optimal enzyme conformations required for thermal resilience.^59,60^ In contrast, variations in enzyme loading, even at high immobilization densities (51.9 mg·g_support_^–1^), did not lead to measurable effects on the thermal stability within the tested range. This indicates that the conformational stabilization conferred by the support matrix predominates over potential crowding effects,^57^ at least within the conditions evaluated.

To gain detailed spatial insights into the localization and distribution of the immobilized enzyme within the microcrystalline cellulose matrix, confocal fluorescence microscopy was carried out. Leveraging the intrinsic fluorescence of the FAD cofactor in 8BxHMFO, visualization was achieved without the need for external labeling, preserving the native structure of the biocatalyst. Multiplanar imaging revealed a uniform and homogeneous distribution of the enzyme throughout the porous structure of the microcrystalline cellulose particles, as observed in different focal planes (Figure 3). This spatial uniformity confirms the effective diffusion and attachment of the enzyme across the full depth of the support, which is a desirable feature for heterogeneous catalysis. Such even spatial distribution facilitates consistent substrate accessibility across the matrix and minimizes diffusional gradients, which can otherwise limit reaction efficiency in packed-bed or batch systems.^61,62^ Moreover, this structural uniformity is expected to enhance the operational stability of the system by preventing localized enzyme overloading and associated mass transfer limitations.^63^

**Figure 3:**
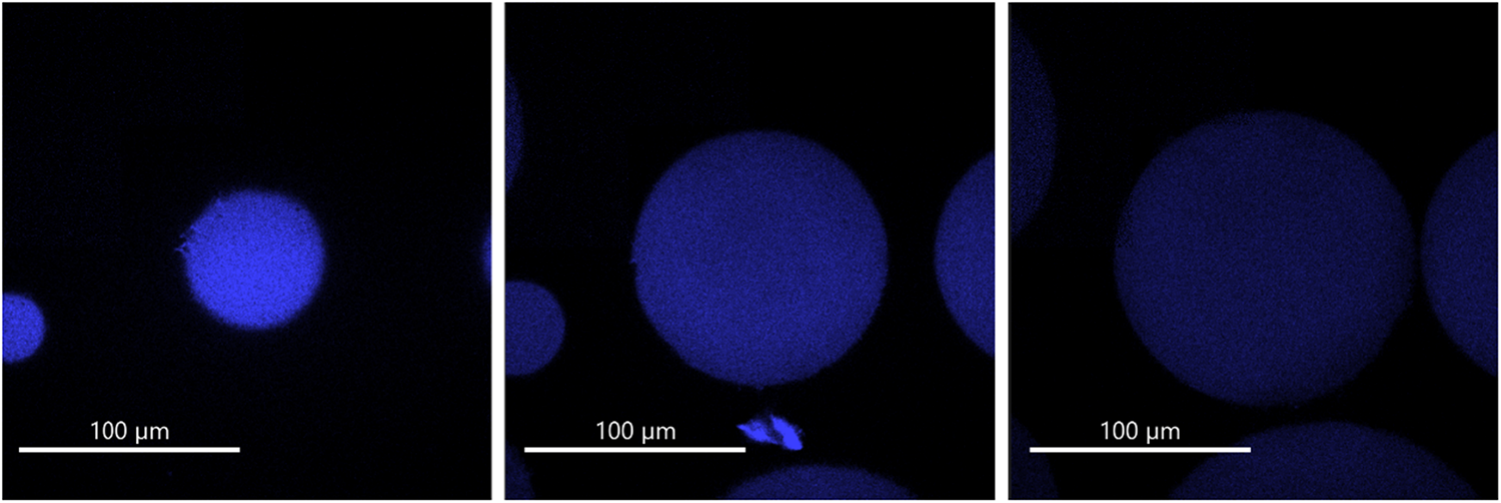
Confocal fluorescence images of three different focal planes of CBM3–8BxHMFO in Perloza MT100 Support.

### Intensification of Enzymatic FDCA Production Using Pure HMF

To assess the catalytic performance of the immobilized CBM3–8BxHMFO system under process intensification conditions, batch reactions were carried out using varying concentrations of pure HMF as the substrate. The reactions were conducted in a 20 mL basket reactor, which maintained a constant air supply 12 mL·min^–1^ (0.6 vvm), temperature 30 °C, and pH 8, scheme of the reaction system is shown in Figure S5. This evaluation aimed to determine the most favorable conditions within the evaluated range for conversion to FDCA, while simultaneously quantifying the accumulation of reaction intermediates and assessing the system productivity.

The enzymatic oxidation of HMF to FDCA was studied over a range of substrate concentrations. Figure 4 focuses on the enzymatic reaction of CBM3–8BxHMFO immobilized on Perloza with pure HMF. It includes the reaction profiles with (A) 6 mM, (B) 12, (C) 24, (D) 50, and (E) 125 mM HMF, as well as (F) the key process metrics (conversion, yield, productivity, and FDCA titer; calculations detailed in the Supporting Information) obtained from these reactions. HMF was rapidly consumed under all tested conditions, and no significant accumulation of intermediates was observed, except in the reaction conducted at 125 mM HMF, where a notable accumulation occurred. In this case, DFF accumulated to approximately 50 mM and persisted throughout the process, indicating inefficient oxidation. Additionally, FFCA, a known potent inhibitor of 8BxHMFO,^22^ also accumulated significantly, reaching concentrations of about 37 mM after 72 h. This accumulation of FFCA correlated directly with the lower FDCA yields observed in this reaction compared with those with lower HMF concentrations, which showed negligible intermediate accumulation and achieved near-complete FDCA yields. It is also important to note that the reaction time increased proportionally with substrate concentration, further influencing productivity at higher HMF levels.

**Figure 4:**
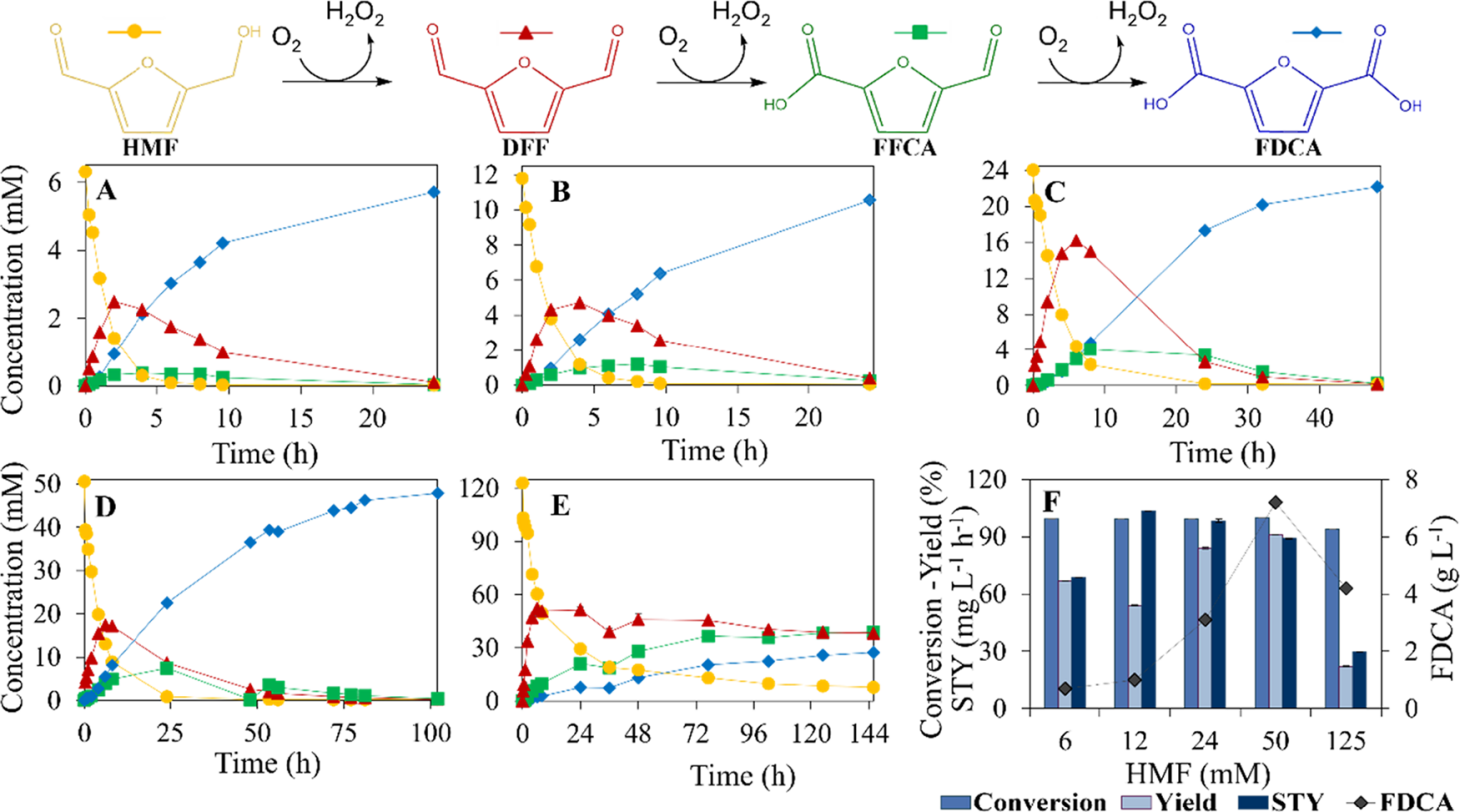
Enzymatic reaction of CBM3–8BxHMFO immobilized on Perloza with pure HMF at 30 °C, pH 8, 500 rpm, 0.6 vvm air bubbling, and catalase was supplemented every 24 h (97.7 U mL^–1^) at different substrate concentrations. Reactions with (A) 6 mM, (B) 12 mM, (C) 24 mM, (D) 50 mM, and (E) 125 mM HMF. (F) Key process metrics of the enzymatic reactions (conversion, yield, productivity, and FDCA titer) obtained with pure HMF at 6, 12, 24, 50, and 125 mM.

From the reactions carried out at different HMF concentrations, key process metrics, such as conversion, yield, and space-time yield (STY) were calculated to identify optimal conditions. The results, summarized in Figure 4F, show that high conversion rates were consistently achieved across all tested concentrations. FDCA yield increased progressively with substrate concentration up to 50 mM, beyond which no significant improvement was observed. In contrast, productivity peaked at 12 mM and showed a slight decline at 24 and 50 mM, with a marked decrease observed for the reaction performed at 125 mM HMF. This behavior is directly related to reaction time; as substrate concentration increases, longer reaction times are required, affecting the productivity. Finally, the total amount of FDCA produced (g·L^–1^) increased proportionally with the substrate concentration, as expected from the direct relationship with yield, reaching a maximum FDCA titer in the 50 mM reaction.

This behavior is consistent with the accumulation of inhibitory intermediates such as FFCA at high HMF concentrations, particularly in the case of the reaction performed at 125 mM HMF, which can adversely affect enzymatic turnover.^22^ Among the evaluated conditions, the reaction with 50 mM HMF provided the highest FDCA titer (7.2 g·L^–1^), representing the best compromise between a high yield and productivity. This performance is notably higher than most enzymatic systems reported to date, which typically reach ≤2 g·L^–1^ FDCA under substrate loadings of 1.5–10 mM HMF using wild-type HMFO or its early variants.^18,19,22^ Even engineered enzymes such as 8BxHMFO generally yielded <2 g·L^–1^ under similar conditions,^22^ while more recent cascades such as BpLac/CglAlcOx achieved only ∼0.8 g·L^–1^ FDCA at 5 mM HMF.^64^ Only very recent advances with alternative enzymes, such as the evolved PedH variant, have approached comparable levels, reaching ∼6 g·L^–1^ from 40 mM HMF.^65^ Notably, although sophisticated systems utilizing immobilized PaoABC have achieved a 100% yield for FDCA production using 100 mM HMF,^66^ they require a complex four-enzyme cocktail, including GOase, PaoABC, HRP, and catalase, to complete the oxidation. In contrast, the current work achieves competitive performance using a single engineered biocatalyst to mediate the entire three-step transformation, thereby significantly reducing the operational complexity and the costs associated with multienzyme preparations.

### Evaluation of Immobilized Biocatalyts under Relevant Industrial/Biorefinery Conditions

To evaluate the system’s applicability under real biorefinery conditions, the reaction was performed using a commercial crude extract (containing 15% of HMF) diluted to a final reaction concentration of approximately 50 mM. Although concentrations up to 125 mM were evaluated with pure substrate, the 50 mM condition was selected for this stage as it corresponded to the highest titer achieved without significant intermediate accumulation. As shown in Figure 5, substrate conversion was nearly complete. DFF levels increased initially but were depleted after 73 h, while FFCA remained at subinhibitory concentrations. The FDCA production profile showed a sustained increase throughout the process, indicating that the biocatalyst remained functionally active, even in the presence of potential impurities present in the crude feedstock.

**Figure 5:**
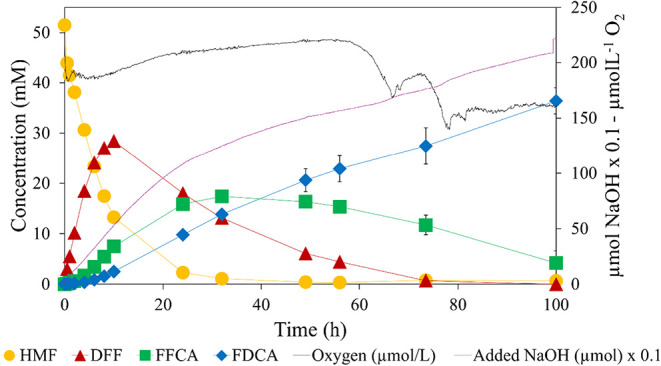
Enzymatic reaction of CBM3–8BxHMFO immobilized on Perloza with 51 mM crude HMF at 30 °C, pH 8, 500 rpm, 0.6 vvm air bubbling and catalase was supplemented every 24 h (97.7 U·mL^–1^).

A comparison between reactions using pure and crude HMF is presented in Table 1. While the immobilized system achieved near-complete conversion in both cases, the FDCA yield decreased from 91.3% (pure) to 70.6% (crude), which consequently impacted the STY.

**Table 1:** Main Results of Enzymatic Reactions with Immobilized CBM3–8BxHMFO at 30 °C, pH 8, and Air Bubbling, Using Crude HMF Compared with Reactions Performed under Same Conditions with Pure HMF

HMF	HMF (mM)	time (h)	conversion (%)	yield (%)	FDCA (g·L^–1^)	STY (mg·L^–1^·h^–1^)
pure	50.6	81	99.7	91.3	7.2	89
crude	51.5	100	98.7	70.6	5.7	57

This decline can likely be attributed to the presence of minor background components in the matrix. HPLC analysis of the crude substrate (Figures S6–S9) allowed for the identification and quantification of furfural (∼180 mM), 4-hydroxybenzaldehyde (∼3.3 mM), and acetic acid (∼20 mM). Furthermore, while these specific inhibitory compounds were identified, the chromatograms indicate that other nonidentified impurities are only present as minor trace peaks. Nonetheless, the enzyme’s ability to sustain FDCA formation in this unpurified technical-grade substrate highlights its robustness and supports its practical application in the context of biorefinery integration and circular economy strategies.

To our knowledge, no reports using enzymes (soluble or immobilized) have previously described the production of FDCA directly from crude HMF. As summarized in Table 2, previous efforts utilizing whole-cell systems or biocatalysis on crude feedstocks generally faced significant limitations. For instance, Birmingham et al.^36^ demonstrated that even the partial oxidation of 1189 mM crude HMF to DFF required extensive enzyme engineering (GOase variants) and biphasic systems to maintain high conversion. Similarly, previous whole-cell studies^67,68^ reported lower FDCA yields (37.2–51.4%) at significantly lower HMF concentrations (8–16 mM), while only a 53.3% yield was achieved with *Klebsiella oxytoca* MCC0144 at 24 mM HMF (Patent WO 2021/124354).^69^ These examples highlight that unrefined feedstocks strongly reduce both yield and achievable product titters.

**Table 2:** Comparison of Biocatalytic Performance for Furanic Production Using Crude or Complex HMF-Containing Feedstocks^a^

References	Catalyst/microorganism	Substrate quality	HMF (M)	Product	Yield (%)	STY (mg·L^–1^·h^–1^)
Yang and Huang	*Burkholderia cepacia* H-2 (whole cells)	algal hydrolysate (spiked)	16	FDCA	51.4^b^	NR
Yang and Huang	*Methylobacterium radiotolerans* G-2 (whole cells)	algal hydrolysate (spiked)	8	FDCA	37.2^b^	NR
Birmingham et al.	GOase M7–2A (free enzyme)	crude/semipurified	1189	DFF	92.0	23,000
Patent WO 2021/124354	*K. oxytoca* MCC0144 (whole cells)	crude (90–99%)	24	FDCA	53.3^b^	20.5
this work	CBM3–8BxHMFO (immobilized enzyme)	crude (15 wt %/wt)	51	FDCA	70.6	57

a NR: Not reported.

b Molar yield calculated from reported mass concentration of product relative to initial substrate concentration.

In comparison, the immobilized CBM3–8BxHMFO maintains high conversion (70.6%) and interesting FDCA titers from 50 mM crude HMF with an STY of 57 mg·L^–1^·h^–1^, outperforming whole-cell systems (Table 2), which typically operate at lower loadings and yields, or require prolonged reaction times to achieve incomplete product formation.^70^ Nonetheless, the gap between pure and crude HMF indicates that further process optimization could enhance the robustness against complex feedstocks and improve productivity. Importantly, our results demonstrate that immobilized CBM3–8BxHMFO is capable of sustaining FDCA production from minimally processed substrates, underscoring the robustness of this biocatalyst and its adaptability to realistic biorefinery streams. These findings strongly support the technical feasibility of integrating this enzymatic platform into waste valorization schemes.

### FDCA Purification and Recovery

In the case of enzymatic FDCA production from crude HMF, a purification process was implemented to isolate and recover the target compound. This step was critical for confirming the feasibility of integrating the biocatalytic platform into real-world applications, particularly in the production of biobased monomers from renewable resources. A scheme of the purification process is shown in Figure S10. Initially, the biocatalyst was removed by filtration, and the FDCA present in the crude reaction mixture was subsequently purified by acid precipitation, washing, redissolution in ethanol, and ethanol removal by distillation. The purified FDCA powder was weighed and dissolved to be analyzed by HPLC. The analysis indicated a purity of approximately 88%, validating the effectiveness of the recovery process but suggesting that minor optimization is needed to reach maximum purity.

As presented in Table S4, the purification strategy allowed for the extraction of 72% of total FDCA into an ethanol-soluble fraction. This high extraction efficiency underscores the environmental compatibility of the process. These results confirm the scalability and technical simplicity of the recovery workflow, while supporting its alignment with sustainable processing standards.

The successful isolation of FDCA from crude lignocellulosic feedstock, coupled with high conversion rates and resilience to impurities demonstrated in the previous section, provides a strong foundation for downstream valorization. These findings reinforce the viability of deploying immobilized oxidases in biorefineries aimed at upgrading biomass-derived intermediates to high-value monomers for the sustainable polymer industry.

### Green Metrics of the Bioprocess

In the context of a carbon-constrained economy, quantifying the environmental impact of chemical and biocatalytic processes is essential to move beyond the qualitative claims of “greenness”. Consequently, the *E*-factor and the global warming potential (GWP) for this study were determined to provide a standardized comparison of waste generation and carbon footprint, based on the approach previously reported.^39^

As previously mentioned, no reports using isolated enzymes have described the production of FDCA directly from crude HMF. Therefore, the comparison of these gate-to-gate green metrics for crude HMF-based FDCA focuses primarily on biotransformations using whole-cell systems. The results, presented in Table 3, demonstrate that the enzymatic process developed in this study is substantially more sustainable than previously reported routes. The data used for determining these metrics are summarized in Table S5.

**Table 3:** Green Metric Values Obtained from Environmental Performance Evaluation for Some Studies and the Present One

*E*-factor	GWP (mass) kg CO_2_-eq·kg_FDCA_^–1^	GWP (energy) kg CO_2_-eq·kg_FDCA_^–1^	references
782.7	70.9	13.31	Yang and Huang
1944.9	175.9	24.78	Yang and Huang
506.4	45.8	36.07	Rode et al.
156.9	15.9	13.02	this study

In the case of the two whole-cell systems,^67,68^ the *E*-factor increases from a 156.9 value obtained in this study to 782.7 and 1944.9; corresponding to 5.0- and 12.4-fold increase in waste generation, highlighting that the yield of the process has a lot of impact in this kind of metrics. Moreover, through the present work, GWP_mass_ is lowered 4.5- and 11.0-fold (70.9 and 175.9 vs 15.9 kg CO_2_-eq·kg_FDCA_^–1^) indicating again that final titers values have a substantial effect on these environmental performance indicators. In terms of GWP_energy_, the value obtained remains nearly the same in both studies (13.02 vs 13.31 and 24.78 kg CO_2_-eq·kg_FDCA_^–1^), indicating that the major sustainability gains arise from conversion and substrate loading, even using higher reaction temperatures.

Finally, compared with the patented biotransformation route using resting cells of *K. oxytoca* (Rode et al.), previously the highest titer reported for crude HMF, at 1.97 g·L^–1^, the enzymatic process in the present study still achieves a 3.2-fold reduction in the *E*-factor and a 2.9-fold and 2.8-fold decrease in GWP_mass_ and GWP_energy_, respectively. This highlights the benefit of intensifying biocatalytic systems directly on crude HMF streams.

It should be noted that these comparisons cover only the reaction (upstream) stage, as downstream operations could not be consistently evaluated due to the lack of harmonized data, and thus the overall environmental advantage of the present process is likely underestimated.

## Conclusion

This work demonstrates the enzymatic production of FDCA from lignocellulosic-derived HMF as a sustainable strategy aligned with circular economy principles. The fusion of 8BxHMFO with CBM3 enabled one-step purification and immobilization on microcrystalline cellulose, resulting in high immobilization yields and improved stability. The biocatalyst showed optimal performance at 50 mM HMF, producing 7.2 g·L^–1^ of FDCA with nearly complete yield. Importantly, the system efficiently converted crude HMF, achieving 98.7% conversion and 70.6% yield, outperforming whole-cell systems that generally exhibit lower titers and longer reaction times. This highlights the robustness of the process against impurities present in lignocellulosic hydrolysates and confirms its relevance under biorefinery conditions. Downstream recovery provided 88% FDCA purity using ethanol as a green solvent, reinforcing the environmental value of the approach. Future efforts should focus on enhancing the recyclability of the biocatalyst, potentially through the coimmobilization of catalase to mitigate oxidative stress, thereby establishing a fully robust industrial platform for biobased polymer production. Overall, these findings, along with the evaluation of sustainable metrics, validate biocatalysis as a sustainable and scalable route for FDCA production from minimally processed substrates, paving the way for future industrial applications.

### Declaration of Generative AI and AI-Assisted Technologies in the Writing Process

During the preparation of this work, the authors used ChatGPT (OpenAI, San Francisco, USA) in order to improve the readability and clarity of the manuscript. After using this tool, the authors reviewed and edited the content as needed and took full responsibility for the content of the published article.

## Supplementary Material



## Data Availability

The data supporting this study are openly available in the CORA repository at https://dataverse.csuc.cat/dataset.xhtml?persistentId=doi:10.34810/data2637.

## Notes

Protein amino acid sequences; FDCA production; CBM3–8BxHMFO immobilization; catalase stability; enzymatic reaction of CBM3–8BxHMFO immobilized on Perloza; melting temperature values; reactor setup; HPLC chromatogram of crude HMF; purification step samples; schematic representation of the FDCA purification process; and reaction parameter equations (PDF)
