# Supplementary material for: Immobilized Hydroxymethylfurfural Oxidase Enables Robust Biocatalytic Production of 2,5-Furandicarboxylic Acid from Crude 5‑Hydroxymethylfurfural
Source: ACS Sustain Chem Eng. 2026 Apr 4;14(15):7205–16. doi: 10.1021/acssuschemeng.5c13549 (PMC13103917; doi:10.1021/acssuschemeng.5c13549)
Supplement: Supplementary file 1 [file sc5c13549_si_001.pdf]

Supporting Information for

# IMMOBILIZED HYDROXYMETHYLFURFURAL OXIDASE ENABLES ROBUST BIOCATALYTIC PRODUCTION OF 2,5-FURANDICARBOXYLIC ACID FROM CRUDE 5-HYDROXYMETHYLFURFURAL

*Darly Concha †, Garazi Ortiz-Orruño †, Marina Guillén †, Oscar Romero †, Kírian Bonet-Ragel † **

† Bioprocess Engineering and Applied Biocatalysis Group, Department of Chemical, Biological and Environmental Engineering, Universitat Autònoma de Barcelona, 08193 Bellaterra, Spain.

**Corresponding Author**

*** Kírian Bonet-Ragel**

Department of Chemical, Biological and Environmental Engineering, Universitat Autònoma de Barcelona, 08193 Bellaterra, Catalonia, Spain.

Telephone: +34 93 581 4791

Kirian.bonet@uab.cat

Supporting Information: 13 pages, 10 figures, 5 tables and 4 equations

**Amino acid sequences**

CBM3:

MNLKVEFYNSNPSDTTNSINPQFKVTNTGSSAIDLSKLTLRYYYTVDGQKDQTF
WCDHAAIIGSNGSYNGITSNVKGTFVKMSSSTNNADTYLEISFTGGTLEPGAHV
QIQGRFAKNDWSNYTQSNDYSFKSASQFVEWDQVTAYLNGVLVW

Linker:

SAGSSAAGSGSG

8BxHMFO:

MTDTIFDYVIVGGGTAGSVLANRLSARPENRVLLIEAGIDTPENNIPPEIHDGLRP
WLPRLSGDKFFWPNLTVYRAAEHPGITREPQFYEQGRLLGGGSSVNMVVSNRG
LPRDYDEWQALGADGWDWQGVLPYFIKTERDADYGDDPLHGNAGPIPIGRVD
SRHWSDFTVAATQALEAAGLPNIHDQNARFDDGYFPPAFTLKGEERFSAARGY
LDASVRVRPNLSLWTESRVLKLLTTGNAITGVSVLRGRETLQVQAREVILTAGA
LQSPAILLRTGIGPAADLHALGIPVLADRPGVGRNLWEHSSIGVVAPLTEQARA
DASTGKAGSRHQLGIRASSGVDPATPSDLFLHIHADPVSGLASARFWVNKPSST
GWLKLKDADPFSYPDVDFNLLSDPRDLGRLKAGLRLIKHYFAYPSLAKYGLAL
ALSRFEAPQPGGPLLNDLLQDEAALERYLRTNVGGVFHASGTARIGRADDSQA
VVDKAGRVYGVTGLRVADASIMPTVPTANTNLPTLMLAEKIADAILTQA* GS

**FDCA production using soluble CBM3-8BxHMFO**

Enzymatic reactions with CBM3-8BxHMFO were carried out using 2.5 µM of enzyme, 6 mM HMF as substrate, and catalase in excess (10–25 U·mL$^{-1}$). Reactions were performed at pH 8.0 and 30 °C while continuously aerating the solution with air at a flow rate of 9 mL·min$^{-1}$. After two days of reaction, an additional dose of HMF (6 mM) and catalase (10–25 U·mL$^{-1}$) was supplied under the same conditions. Aliquots were collected daily to determine the residual enzymatic activity. Substrate consumption, intermediate accumulation, and FDCA formation were quantified by HPLC.

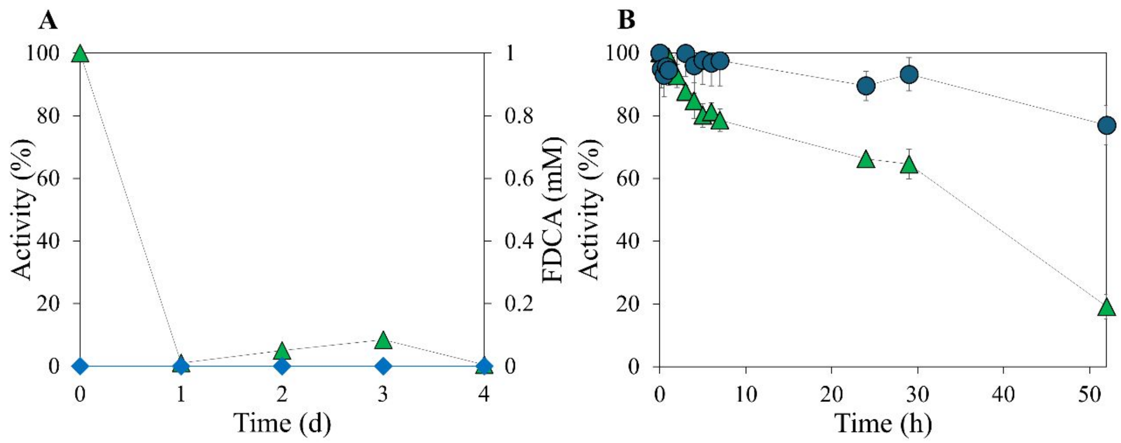


**Figure S1**. A) FDCA production with 2.5 µM CBM3-8BxHMFO and 6 mM HMF at pH 8, 30 °C, 500 rpm, 0.6 vvm air. FDCA (blue diamonds), residual activity (green triangles). B) Enzyme (CBM3-8BxHMFO) stability at pH 8 and 30 °C with air 0.6 vvm (green triangles) and without sparging (blue dots).

**Table 1S**: Immobilizations of CBM3-8BxHMFO on microcrystalline cellulose.

| Support | Avicel® PH-200 | Perloza MT-100 medium |
|---|---|---|
| Offered activity ($U \cdot g^{-1}_{support}$) | 4.28 | 3.73 |
| Immobilization yield (IY%) | 92.7 | 91.7 |
| Recovered activity (RCA%) | 27.2 | 28.8 |
| Immobilized protein ($mg \cdot g^{-1}_{support}$) | 48.0 | 51.9 |

### Catalase Activity Assay

Catalase activity was determined spectrophotometrically by monitoring the decomposition of hydrogen peroxide ($H_2O_2$) at 240 nm ($\varepsilon_{240}$ = 0.0436 $mM^{-1}$ $cm^{-1}$). One unit (U) of catalase activity was defined as the amount of enzyme required to catalyze the degradation of 1 µmol of $H_2O_2$ per minute under the specified assay conditions.

Standard measurements were performed at 30 ºC in 3 mL quartz cuvettes using a Varian Cary 50 UV-Vis spectrophotometer (Agilent Technologies). The reaction mixture comprised 2000 µL of 0.036% (w/w) $H_2O_2$ dissolved in 50 mM Tris-HCl buffer (pH 8.0) and 100 µL of the enzymatic solution.

Additionally, the assay was adapted for microscale analysis using UV-transparent multiwell plates in a SPECTROstar Nano plate reader (BMG LABTECH GmbH, Germany). For the microplate format, the reaction volume was scaled down to 190 µL of the aforementioned $H_2O_2$ substrate mixture and 10 µL of the enzymatic solution. Calculations for the microplate assay were adjusted using an optical pathlength of 0.62 cm. All activity measurements were performed in triplicate, and appropriate blank corrections were applied to account for non-enzymatic $H_2O_2$ degradation.

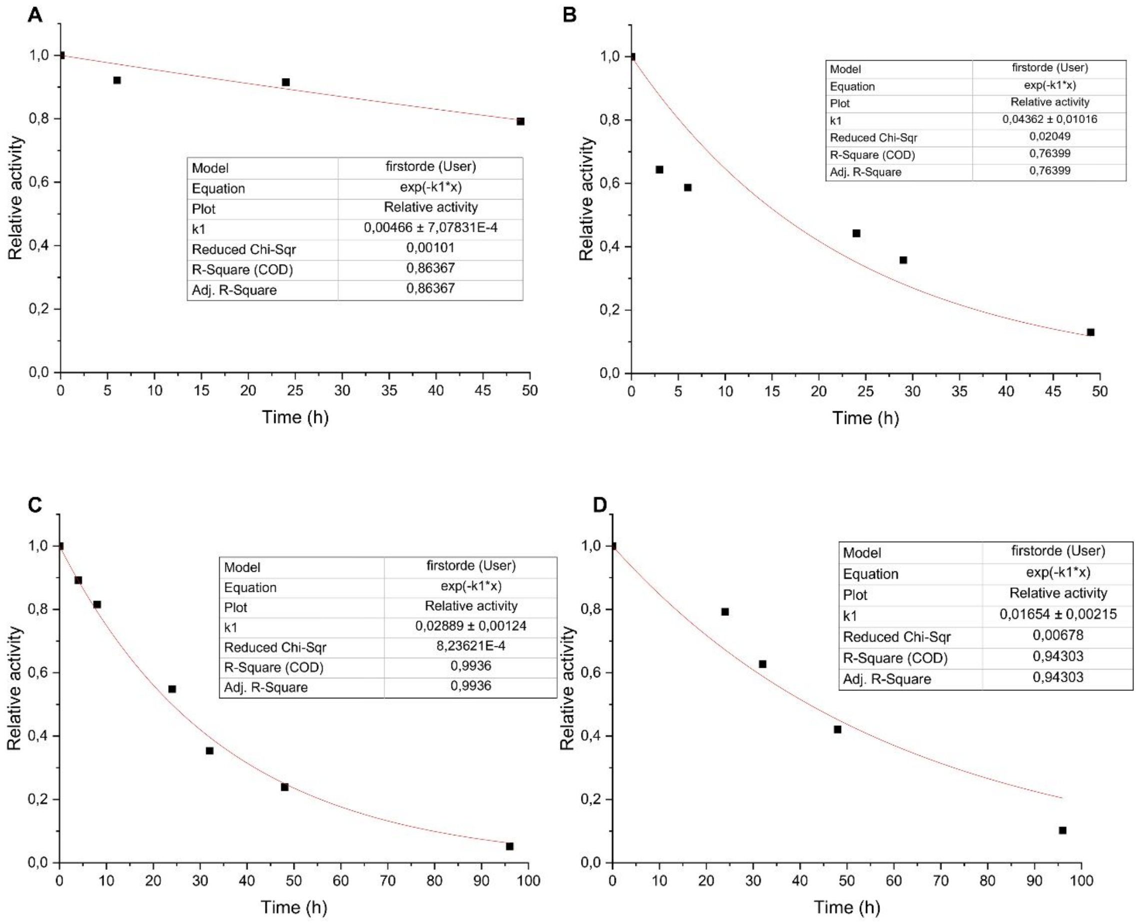


**Figure S2**. Thermal and operational stability of catalase at pH 8.0 and 30°C, fitted to a first-order inactivation kinetics. (A) Stability in Tris-HCl buffer without aeration ($k_1$ = 0.00466 $h^{-1}$; $t_{1/2}$ = 148.7 h). (B) Stability in Tris-HCl buffer under constant aeration (0.6 vvm) ($k_1$ = 0.04362 $h^{-1}$; $t_{1/2}$ = 15.9 h). (C) Stability in 50 mM HMF solution ($k_1$ = 0.02889 $h^{-1}$; $t_{1/2}$ = 24 h). (D) Stability in 50 mM crude HMF solution ($k_1$ = 0.01654 $h^{-1}$; $t_{1/2}$ = 41.9 h).

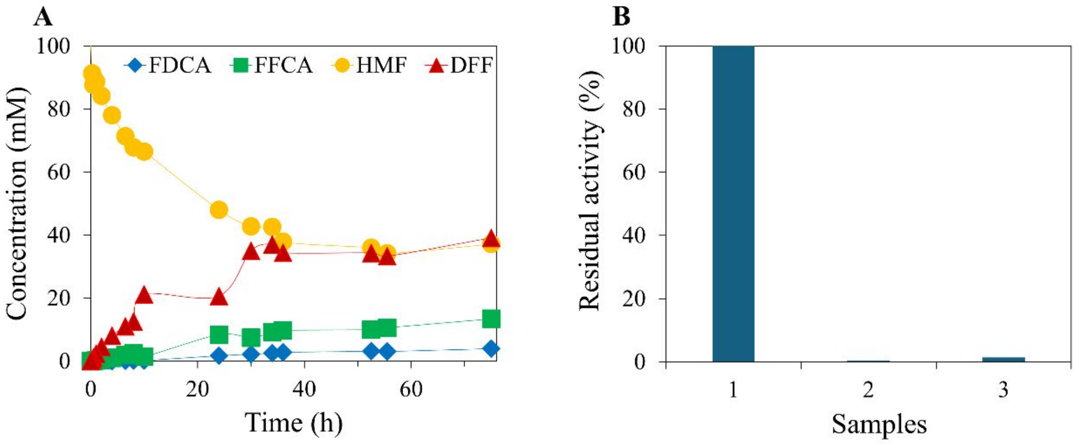


**Figure S3**. Enzymatic reaction of CBM3-8BxHMFO immobilized on Perloza with 100 mM HMF at 30 °C, 500 rpm, pH 8, and 0.6 vvm air sparging. A) Concentration profiles of HMF, intermediates (FFCA and DFF), and the product FDCA. B) Residual activity of samples collected at different stages: (1) initial sample, (2) final sample, and (3) final reaction supernatant

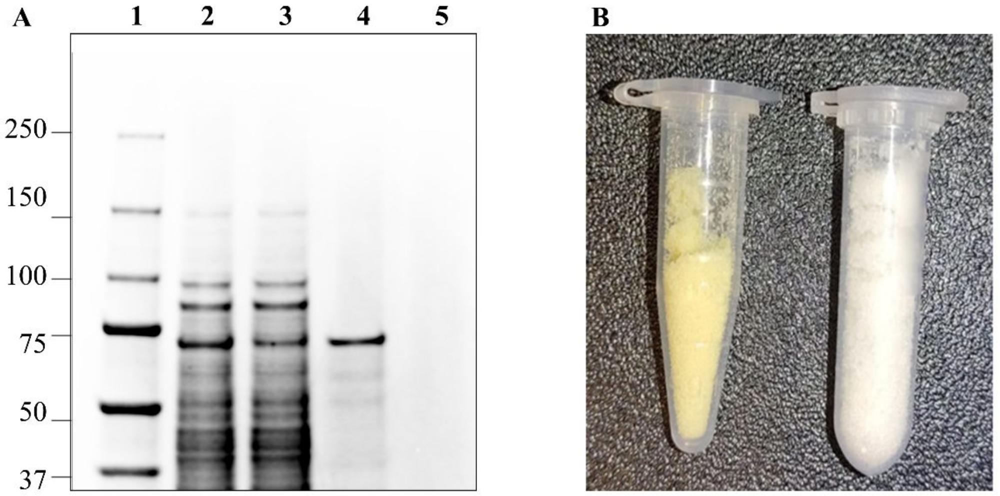


**Figure S4**. (A) SDS-PAGE gel of immobilization and enzymatic reaction fractions. Lane 1: molecular weight marker (kDa); CBM3-8BxHMFO, 75.1 kDa. Immobilization on Perloza: lanes 2–5 correspond to blank (Total lysate), supernatant (Lysate after immobilization), immobilized derivative before the reaction, and immobilized derivative after the reaction, respectively. (B) Immobilized derivative CBM3-8BxHMFO on Perloza before and after the enzymatic reaction with 100 mM HMF.

**Table S2**. Protein content in the biocatalyst before and after the enzymatic reaction.

| | Total protein (immobilized derivative) (mg) | Protein (%) |
|---|---|---|
| Before | 104,1 | 100 |
| After | 3,9 | 3,8 |

**Table S3**. Melting temperature values of soluble and immobilized CBM3-8BxHMFO. Low load (8.1 $mg \cdot g^{-1}_{support}$), high load (51.9 $mg \cdot g^{-1}_{support}$) .

| | Glutaraldehyde (%) | $T_m$ (°C) |
|---|---|---|
| Soluble | 0 | 49.7 ± 0.24 |
| Immobilized biocatalyst (low load) | 0 | 52.5 ± 0.00 |
| | 0.25 | 51.5 ± 0.26 |
| Immobilized biocatalyst (high load) | 0 | 53.2 ± 0.29 |
| | 0.25 | 52.0 ± 0.00 |

A

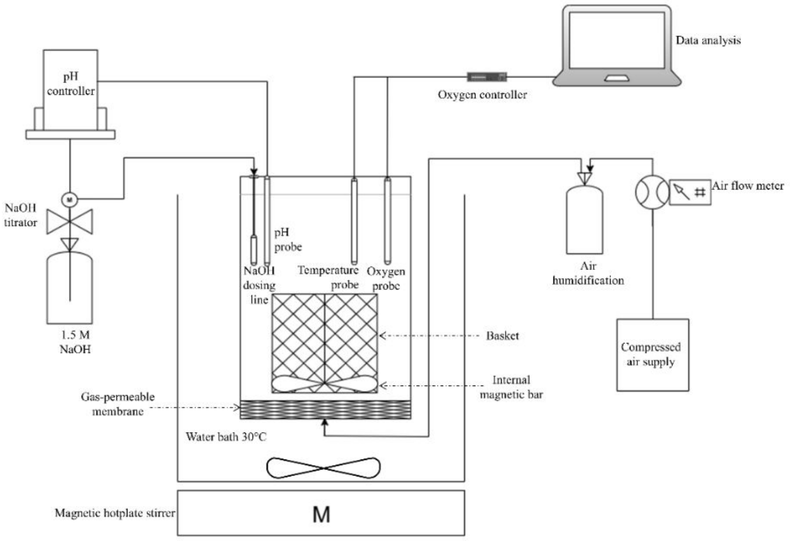


B

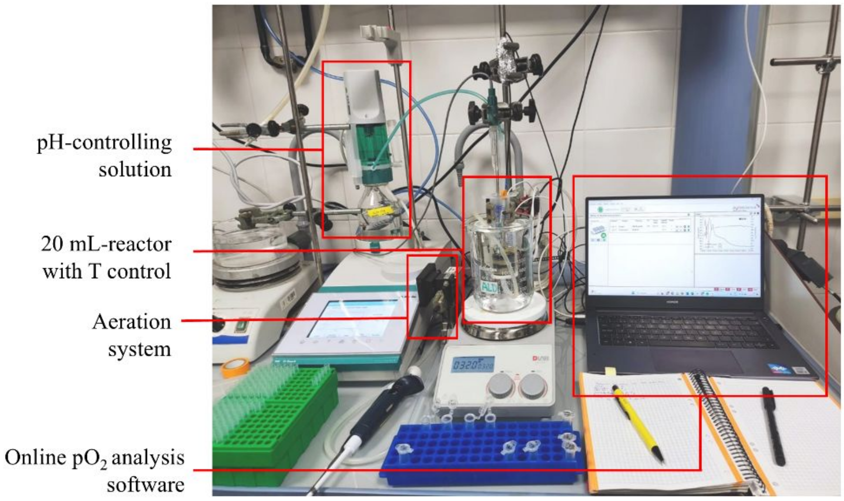


**Figure S5**: Reactor setup for the enzymatic production of FDCA. (A) Schematic representation of the 20 mL reactor configuration, including pH and temperature control, aeration system, and online $pO_2$ monitoring. (B) The experimental setup with the main components highlighted.

## HPLC analysis of crude HMF

Diode-array detection (DAD)

Spectral scanning and impurity profiling of the crude HMF were performed on an Agilent 1100 Series HPLC system equipped with an autosampler, a thermostated column compartment, and an online DAD. Separation was carried out on an ion-exchange SUPELCOGEL C-610H column at 30 °C. The mobile phase was 5 mM $H_2SO_4$ at a flow rate of 1.0 mL/min. Spectral data were recorded across a 200–900 nm wavelength range, with specific extraction at 264 nm.

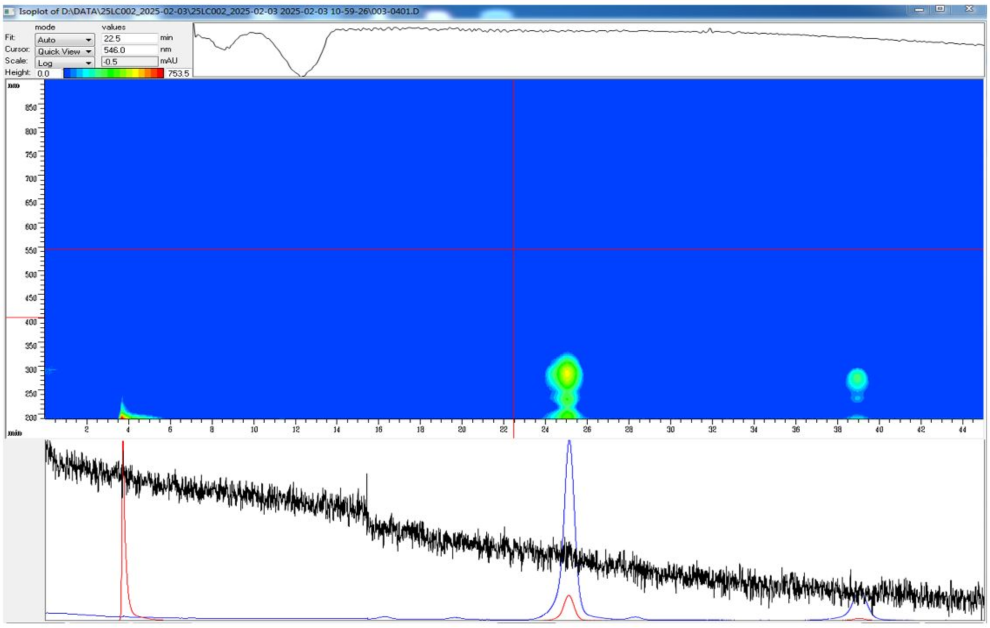


**Figure S6**: HPLC chromatogram of crude HMF recorded with diode-array detection (200–900 nm). Spectral scan across wavelengths showing absorbance distribution of major peaks.

Furanic derivatives 1: HMF, FDCA, FFCA, HMF, DFF and Fulfural

To optimize the separation of specific furanic derivatives and furfural, the flow rate on the standard Agilent 1220 Infinity system (described in the main text) was increased to 1.0 mL/min using the same SUPELCOGEL C-610H column and 5 mM $H_2SO_4$ mobile phase at 30 °C. Detection was maintained at 264 nm. Under these modified high-flow conditions, the retention times shifted accordingly to accommodate the separation of furfural alongside the main oxidation products.

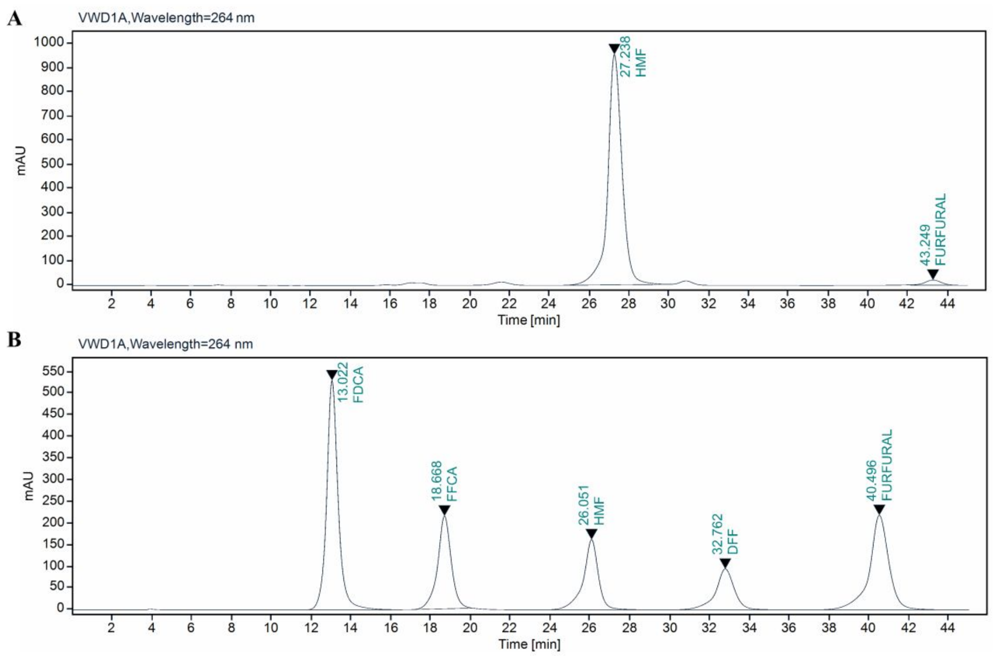


**Figure S7**: HPLC chromatograms of crude HMF and furanic derivatives. A) Crude HMF showing the main compounds (HMF and furfural). B) Furanic standards containing FDCA, FFCA, HMF, DFF (2,5 mM), and furfural (4 mM). Detection was carried out at 264 nm.

Furanic derivatives 2: HMF, FFCA, furfural, 4-HBA, vanillin and syringaldehyde

Chromatographic analyses were performed on an Agilent 1220 Infinity system (described in the main text) equipped with a Waters CORTECS C18+ column (4.6 x 150 mm, 2.7 µm particle size). The detection wavelength was set at 210 nm. The mobile phase comprised 0.1% (v/v) aqueous trifluoroacetic acid (eluent A) and acetonitrile (eluent B). Gradient elution was applied at a constant flow rate of 1.0 mL/min according to the following program: an initial linear gradient from 10% to 40% eluent B over 15 min, followed by a second linear gradient from 40% to 60% eluent B up to 20 min. Finally, the solvent composition was linearly reverted to the initial conditions (10% eluent B) over 5 min (from 20 to 25 min) for column re-equilibration prior to the subsequent injection.

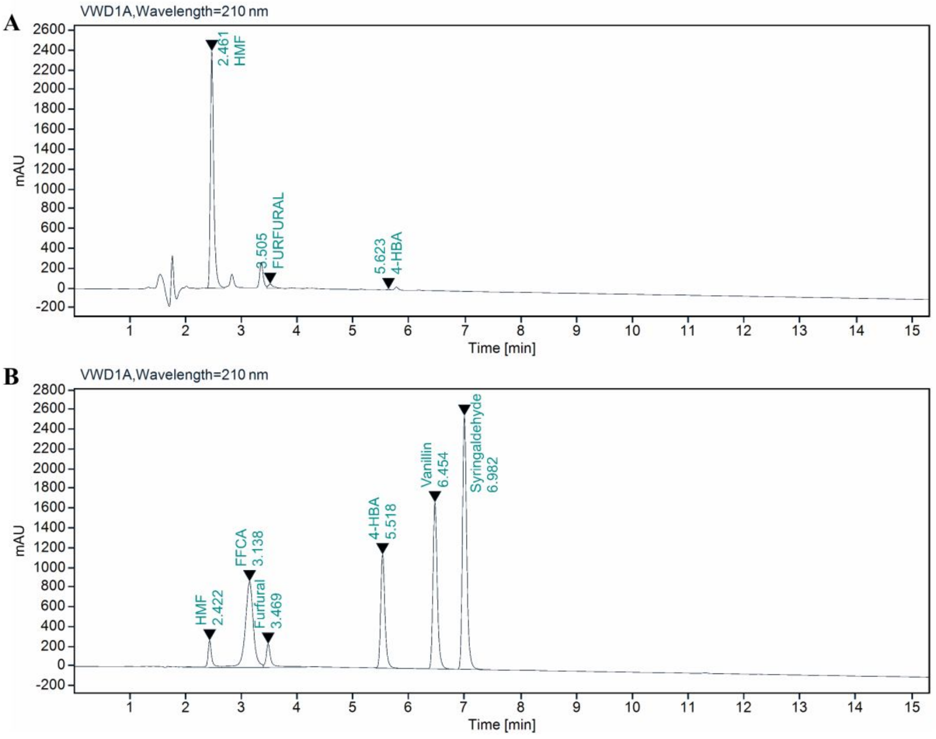


**Figure S8:** HPLC chromatograms of crude HMF and furanic derivatives, eluted with a gradient of 0.1% TFA and acetonitrile at a flow rate of 1 mL/min at 30ºC, using a C18 column, and detected at 210 nm. A) Crude HMF showing the main compounds (HMF, furfural and 4-hydroxybenzaldehyde (4-HBA)). B) Standards mix containing HMF, FFCA, furfural, 4-HBA, vanillin and syringaldehyde (1.5 mM).

Sugars and organic acids

The identification and quantification of organic acids, specifically acetic acid, were conducted using a Thermo Scientific Dionex Ultimate 3000 HPLC system (equipped with a refractive index (RI) detector. The analysis utilized a Coregel 87H3 ion-exclusion column at 25 °C. The mobile phase consisted of 5.7 mM $H_2SO_4$ delivered at a flow rate of 0.6 mL/min.

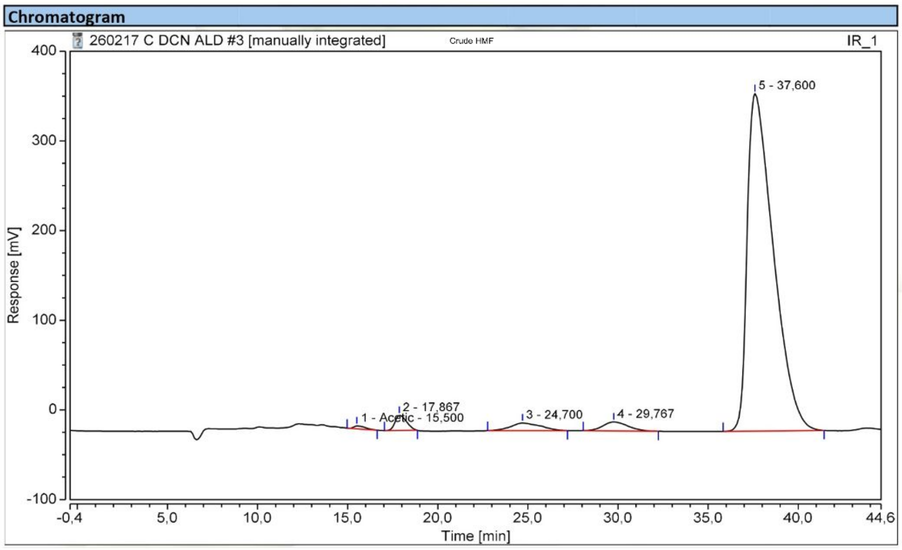


**Figure S9:** HPLC chromatograms of crude HMF, eluted with 5.7 mM $H_2SO_4$ at a flow rate of 0.6 mL/min at 25ºC, using a  Coregel 87H3 ion-exclusion column, and detected by refractive index (RI), showing the main compounds HMF, and acetic acid.

**Table S4**: Different samples from purification process FDCA.

| Samples | FDCA (mM) | Volume (mL) | FDCA (μmol) | FDCA (%) |
|---|---|---|---|---|
| Final reaction solution | 33.9 | 17.8 | 600.7 | 100 |
| Precipitate supernatant | 5.8 | 19.8 | 114.1 | 19 |
| Wash acid water | 0.9 | 53.3 | 45.2 | 7.5 |
| Solubilized in ethanol | 8.1 | 53.3 | 432.2 | 72 |

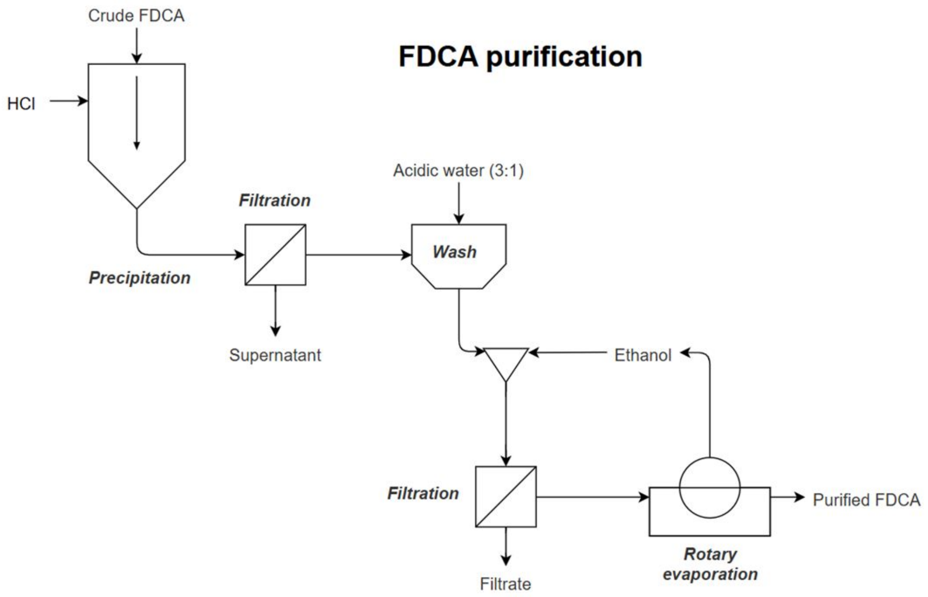


**Figure S10**: Schematic representation of the FDCA purification process following enzymatic conversion using crude HMF.

**Table S5**. Raw data used for GWPs and E-factor calculations.

| % water | SL (kg ·L$^{-1}$) | Conv (%) | ΔT (ºC) | Product mass (kg) | Waste mass (kg) | References |
|---|---|---|---|---|---|---|
| 100 | 0.002 | 51.5 | 8 | 0.00016 | 0.12983 | Yang, 2016 |
| 100 | 0.001 | 41.5 | 6 | 0.00002 | 0.03997 | Yang, 2018 |
| 100 | 0.003 | 53.1 | 10 | 0.00009 | 0.04990 | Rode, 2021 |
| 100 | 0.006 | 70.6 | 10 | 0.00014 | 0.01785 | This study |

## Calculation of Reaction Parameters

The catalytic performance of the immobilized system was evaluated based on the consumption of the substrate (HMF) and the formation of the target product (FDCA). The key process metrics were calculated as follows:

$$Conversion\ (\%) = \frac{[HMF]_i - [HMF]_f}{[HMF]_i}\ x\ 100$$ Equation S1

where $[HMF]_i$ is the initial substrate concentration (mM) and $[HMF]_f$ is the residual substrate concentration at final time.

$$FDCA\ Yield\ (\%) = \frac{[FDCA]_f - [FDCA]_i}{[HMF]_i}\ x\ 100$$ Equation S2

where $[FDCA]_f$ is the concentration of the product formed (mM), [FDCA]i is the initial concentration of the product (mM), and $[HMF]_i$ is the initial substrate concentration (mM).

$$FDCA\ Titer\ (g\ L^{-1}) = [FDCA]\ x\ MW_{FDCA}\ x\ 10^{-3}$$ Equation S3

where $MW_{FDCA}$ is the molecular weight of 2,5-furandicarboxylic acid (156.09 $g{\cdot}mol^{-1}$) and [FDCA] is the product concentration in mM.

$$STY\ (mg\ L^{-1}h^{-1}) = \frac{Titer\ x\ 1000}{t}$$ Equation S4

where Titer is the product concentration ($g{\cdot}L^{-1}$) and t is the reaction time (h).
